# Defining the Standard of Care in Fournier’s Gangrene—An Umbrella Review

**DOI:** 10.1016/j.euros.2025.11.009

**Published:** 2025-12-03

**Authors:** Charlotte Müssgens, Laura Wimmer, Christian Daniel Fankhauser, Laila Schneidewind, Fabian Joel Aschwanden

**Affiliations:** a Department of Urology, University Hospital Bern, Inselspital, University of Bern, Bern, Switzerland; b Department of Urology, Luzerner Kantonsspital, University of Lucerne, Lucerne, Switzerland

**Keywords:** Urological infections, Fournier’s gangrene, Necrotizing fasciitis, Sepsis, Debridement

## Abstract

**Background and objective:**

Fournier’s gangrene (FG) is a life-threatening bacterial infection. Unfortunately, outcomes have not improved in recent years mainly due to the fact that robust research is challenging in this very rare disease. Consequently, we conducted an umbrella review regarding the standard of care for FG to provide best evidence for clinicians and identify research gaps to plan high-quality studies.

**Methods:**

The recommendations provided in the *Cochrane Handbook of Systematic Reviews* and the Preferred Reporting Items for Systematic Reviews and Meta-analyses guidelines were followed. The full review protocol was pre-registered and is available at PROSPERO (CRD 42023489596).

**Key findings and limitations:**

The primary literature search yielded 3366 references. Finally, we were able to include seven systematic reviews including two meta-analyses. Up to 40% of patients may initially lack cutaneous signs, risking a diagnostic delay and progression to high-mortality disease. Diabetes mellitus emerged as the most prevalent comorbidity across the reviews (32–66%) and was most frequent among nonsurvivors. The management of FG is consistently described as a multimodal emergency, centered on three core interventions: early surgical debridement, empiric broad-spectrum antibiotics, and intensive supportive care. Adjunct interventions need further evaluation. Despite advances in multimodal care, FG carries high mortality and morbidity. There is only moderate confidence mainly due to the risk of bias from the individual studies being included in the reviews.

**Conclusions and clinical implications:**

Across the seven reviews, FG remains a life-threatening condition with substantial mortality. Further comprehensive research is needed desperately; a prospective registry might be suitable for managing this rare disease.

**Patient summary:**

We conducted an umbrella review, a review including only systematic reviews and meta-analyses, about the standard of care for Fournier’s gangrene (FG), which is a life-threatening and rare bacterial infection, and the clinical outcomes have not improved in recent years. In summary, FG is consistently described as a multimodal emergency, centered on three core interventions: early surgical debridement, empiric broad-spectrum antibiotics, and intensive supportive care; however, despite advances in care, FG is still associated with high mortality and morbidity. Consequently, further robust research is needed.

## Take Home Message

The literature emphasizes the need for early intervention, risk stratification tools, and standardized outcome reporting to guide future clinical decision-making for Fournier’s gangrene, but further high-quality research is essential. In this context, a prospective registry study might be most suitable.

## 1 Introduction

Fournier’s gangrene (FG) is a sporadic, life-threatening, necrotizing, bacterial infection affecting the perineum, perineal region, and genitals [1], [2], [3], [4]. Unfortunately, the incidence of this disease is very low, and so there is limited knowledge about FG and its standard of care. The data are arising mostly from retrospective case series with small sample sizes [4], [5], [6], [7], [8], [9]. Furthermore, the prognosis, survival, and outcome of FG have not improved in recent years despite more intensive critical-care therapy for these patients [3], [4], [10]. However, the key points for successful treatment of FG are immediate surgical debridement, accompanied by intensified antibiotic therapy and intensive care medical management, but mortality still remains high [4], [11]. Further research is needed to improve outcomes for FG. In addition, a detailed definition of the standard of care is necessary, especially given that there is currently no standardized approach to the diagnosis, treatment, or long-term management of FG across Europe [4].

Interestingly, Schneidewind et al. [4] reported in their European survey of FG that the biggest challenges associated with FG are the short time to diagnosis and treatment, standardization and establishment of guidelines, as well as disease awareness.

Consequently, we conducted a systematic review with the primary aim to evaluate and summarize the recent standard of care for FG, regarding diagnosis and treatment. This is an umbrella review including only systematic reviews and meta-analyses to provide the best evidence.

## 2 Methods

### 2.1 Search strategy and study inclusion/exclusion criteria

We followed the recommendations provided in the *Cochrane Handbook of Systematic Reviews* [12] and the Preferred Reporting Items for Systematic Reviews and Meta-analyses (PRISMA) guidelines [13]. Thus, in April 2024, we performed a systematic literature search using MEDLINE via PubMed and the Cochrane Library database. The search algorithm broadly included the following search term clusters: “Fournier’s gangrene,” “necrotizing fasciitis,” “cellulitis,” “perineum,” and “genitalia.” Reference lists of the included reviews were searched to identify additional records. No restrictions were made concerning language or study region. Studies published after January 2000 were included, as this marks for modern intensive care treatment. This paper will include only systematic reviews, and it was prospectively registered at PROSPERO (https://www.crd.york.ac.uk/prospero/; ID CRD 42023489596).

We included adult patients undergoing any kind of treatment for diagnosed FG. No treatment or exposure restrictions were applied, and no comparator was predefined to gain all the available knowledge. The full review protocol is published in PROSPERO.

### 2.2 Data extraction

An a priori defined standardized data extraction process was used for every included record. The extracted variables included author(s), year of publication, study country, population size, percent of female patients, risk factors for the development of FG and worse outcome, diagnostic measures, as well as microbiological aspects of FG and all details of treatment. Study extraction was performed independently by two review authors (L.W. and F.J.A.). Inconsistencies were resolved by a third review author (L.S.). The online platform Covidence (https://www.covidence.org/; Veritas Health Innovation Ltd, Melbourne, Australia) was used for the screening and data extraction process.

### 2.3 Study quality assessment

For quality assessment, the AMSTAR2 tool was used. It was designed to evaluate the quality of systematic reviews including the following critical domains: protocol registered before commencement of the review, adequacy of the literature search, justification for excluding individual studies, risk of bias from individual studies being included in the review, appropriateness of meta-analytical methods, consideration of the risk of bias when interpreting the results of the review, and assessment of the presence and likely impact of the publication bias. This leads to overall rating of confidence in the results of the review (high, moderate, low, and critically low) [14]. Risk of bias assessment was performed by two independent review authors (C.M. and L.S.), and any disagreement was resolved by a third author (F.J.A.).

### 2.4 Evidence synthesis

As we did not expect sufficient homogeneity between the studies, the conduction of a meta-analysis was not planned. Descriptive evidence synthesis and tables will be used to present study characteristics, for example, study design, sample sizes, interventions, or outcomes.

## 3 Results

**Fig. 1:**
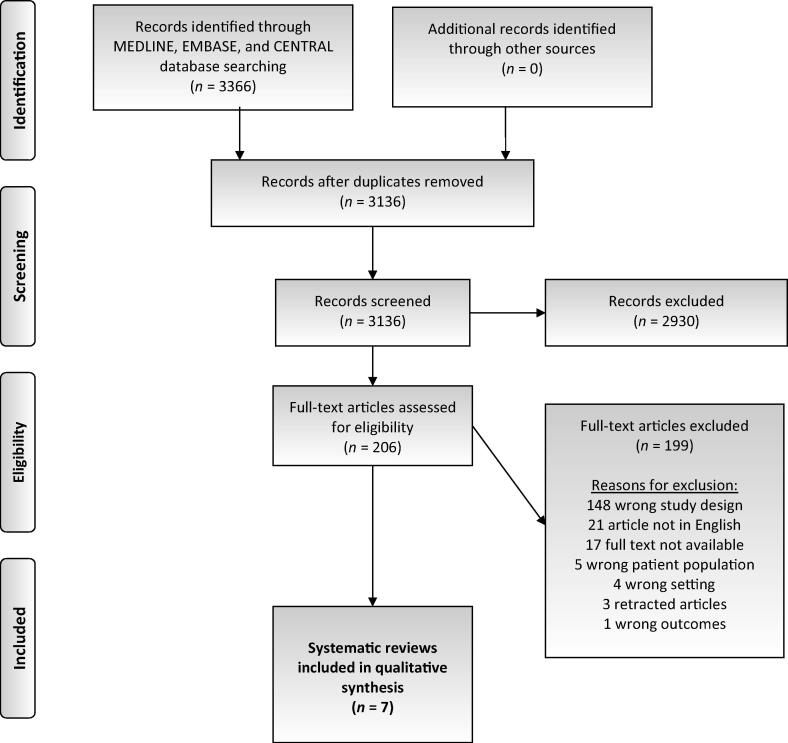
PRISMA flowchart of the study selection process. PRISMA = Preferred Reporting Items for Systematic Reviews and Meta-analyses.

An umbrella review was conducted to define the standards in the diagnosis and treatment of FG. The primary literature search yielded 3366 references. Finally, we were able to include seven systematic reviews, including two meta-analyses [15], [16], [17], [18], [19], [20], [21]. The PRISMA flowchart of the study selection process is illustrated in Fig. 1. Table 1 shows the main characteristics as well as the findings of the included studies.

**Table 1:** Main characteristics and findings from the included systematic reviews (*n* = 7)

Reference	Review design	Included studies/population	Results Diagnostics and risk factors	Results Treatment	Results Outcomes	Authors’ conclusions
Azmi et al. (2025) [15]	Systematic review of case reports	14 Case reports included Male to female ratio 10:4 Aged between 34 and 72 yr 10 obese patients	Male sex, middle aged (median: 55 yr old), obese, and long-term type 2 diabetes as prevalent among FG cases in SGLT2i users	–	Favorable recovery after prompt drug cessation and multimodal standard treatment; no clear definition of FG given	The incidence of FG following SGLT2i use is rare The patient’s outcome improved after multimodal treatment and replacing SGLT2i with other antidiabetic drugs
Shet et al. (2024) [16]	Systematic review	57 studies included, with a median study sample size of 45 participants, encompassing in total 3646 patients	Female sex at a higher risk of death, despite the lower overall incidence of FG in women HIV infection Fungal coinfection Polymicrobial infections (particularly those including *E. Coli* anaerobes, or fungi) were disproportionately represented in fatal cases Comorbidities: diabetes mellitus (most common), vascular disease, and neurological impairment (eg, spinal cord injury), were frequently encountered among nonsurvivors	–	Pooled mortality rate across the cohort was 20.4% Nonsurvivors were older, with a mean age of 61.3 yr, compared with survivors Female patients had a higher mortality rate than male patients (29.8% vs 20.5%) Diabetes as the most prevalent comorbidity among those who died (1162 cases) with a somewhat lower mortality rate (27.5%) than other comorbidities HIV-positive individuals had a strikingly high mortality rate of 54.2% Fungal coinfections (predominantly *Candida* spp.) had the highest mortality rate (68.2%) *E. coli* was the most common pathogen found in fatal cases	Certain demographic (eg, female sex) and clinical (eg, sepsis, fungal involvement) features increase mortality risk markedly, improving survival hinges on prompt risk stratification and initiation of aggressive, targeted treatment
Susini et al. (2024) [17]	Systematic review of original studies aimed at discussing FG reconstruction with at least three clinical cases	38 articles included 719 participants, male to female ratio 478:37 Mean age 51 yr Comorbidities: diabetes mellitus 207 (40.5%), cardiovascular disease 31 (6.0%), obesity 23 (4.5%), smoking 22 (4.3%), alcoholism 21 (4.1%), renal failure 19 (3.7%), immunosuppression 15 (2.5%), cirrhosis 13 (2.5%), paraplegia 6 (1.1%), and neoplasm 6 (1.1%)	Male sex predominance (92.6%) Mean age 51 yr Diabetes mellitus as the most frequently reported comorbidity (40.5%) aligning with broader literature identifying diabetes as a key risk factor for the development and severity of FG	576 reconstructive procedures were performed Minimally invasive techniques (77.6%)—secondary intention skin grafts and local random flaps Locoregional flaps (22.4%)—anatomically based (eg, pudendal thigh flap) No free flaps Complications occurred in 37.8%: wound dehiscence (9.9%), flap necrosis (3.3%), local infection (2.9%), seroma (1.8%), sepsis (1,4%), and hematoma (1.1%) 78.7% of patients experienced no complications after reconstruction	Minimally invasive methods (secondary intention healing, skin grafts, local flaps) were effective in 77.6% of cases, especially when defects were limited in size No deaths were attributed to reconstructive procedures themselves	FG requires immediate medical interventions: broad-spectrum antibiotic therapy, surgical debridement, adjuvant therapies, and reconstructive surgeries. Taking into account, the anatomical characteristics of the inguinal region, skin grafts and local random flaps could offer versatile and effective reconstructions for most FG cases. More invasive strategies should be reserved
Lewis et al. (2021) [18]	Systematic review of literature	37 studies included Male to female ratio: 3093:131 52.7% of the patients had pre-existing comorbidities such as diabetes, IV drug use, liver failure, and immune impairment	Diagnosis is primarily clinical, based on symptoms (scrotal/labial pain, erythema, crepitus, and fever) Up to 40% of patients may lack visible skin signs early on Imaging (CT, US, x-ray) can help assess extent of disease, but should not delay surgical treatment Diabetes mellitus: most common comorbidity Male sex and advanced age are common demographic risk factors Immunosuppression, including HIV Chronic alcoholism and malignancies Delayed surgical debridement strongly associated with increased mortality	Time and extensive debridement play a large role in a better FG prognosis Regarding HBO therapy there is a risk of bias	–	FG necessitates immediate medical attention Most effective treatment protocol for patient survival: administration of broad-spectrum antibiotics along with emergency surgical debridement
Sarofim et al. (2021) [19]	Systematic review and meta-analysis	27 articles with 1482 participants 84% male Age range 47–65 yr	–	Pooled mortality rate was significantly higher in patients who required a stoma (OR 1.71, 95% CI 1.13–2.59, *p* = 0.01) Mean hospital stay was longer in the stoma group but was not statistically significant	–	The use of diversional stoma in FG is a predictor of poor outcomes, but fecal diversion does not reduce the risk of mortality
Schneidewind et al. (2020) [20]	Systematic review	13 studies with a total of 376 participants 202 received HBO therapy and 174 did not	–	–	In five comparative (retrospective case-control) studies encompassing 319 patients, the mortality rate was 16.6% in the HBO group versus 25.9% in the non-HBO group. The overall risk of bias in the included studies was assessed as moderate to high, due to retrospective designs, variability in HBO protocols, and small sample sizes	While evidence is not definitive, HBO therapy has potential as an adjunct in FG treatment, but it is challenging to carry out further studies, mainly due to the rareness of FG and availability of HBO therapy
Silverii et al. (2020) [21]	Meta-analysis of randomized controlled trials	84 studies included 42 415 and 27 158 patients in the SGLT2i and comparator groups, respectively	No significant increase in FG risk in type 2 diabetes mellitus patients treated with SGLT2is No difference was observed between SGLT2is and comparators in the risk of FG (MH-OR 0.41 [0.09, 1.82]), abscess (MH-OR 0.94 [0.54, 1.65]), cellulitis (MH-OR 0.90 [0.71, 1.13], or erysipelas (MH-OR 0.89 [0.45, 1.77])	–	–	No clear increased risk of FG in SGLT2i users based on randomized trial data Caveat: limited total events and wide confidence intervals warrant cautious interpretation

CI = confidence interval; CT = computed tomography; FG = Fournier’s gangrene; HBO = hyperbaric oxygen; HIV = human immunodeficiency virus; IV = intravenous; MMH-OR = Mantel-Haenszel odds ratio; OR = odds ratio; SGLT2i = SGLT2 inhibitor; US = ultrasound.

### 3.1 Diagnosis and risk factors of FG

#### 3.1.1 Diagnosis

FG is primarily diagnosed clinically, with early symptoms including perineal or scrotal pain, erythema, swelling, and crepitus. However, up to 40% of patients may initially lack cutaneous signs, risking diagnostic delay and progression to high-mortality disease. When used, imaging modalities, particularly computed tomography and magnetic resonance imaging, can aid in identifying subcutaneous emphysema and delineating disease extent for further surgical planning, but their use should not delay prompt surgical debridement [18].

#### 3.1.2 Risk factors

Diabetes mellitus emerged as the most prevalent comorbidity across the reviews (32–66%) and was most frequent among nonsurvivors [15], [16], [18], [21]. Other common risk factors included obesity, cardiovascular disease, renal and hepatic impairment, malignancy, and alcohol abuse [16], [17]. Immunocompromised states, especially human immunodeficiency virus (HIV) infection, were associated with the highest mortality rate (54.2%), although data remain limited [16].

Regarding SGLT2 inhibitors (SGLT2is), Silverii et al. [21] found no significant association with FG in a meta-analysis of randomized controlled trials (odds ratio [OR] 0.41, 95% confidence interval [CI] 0.09–1.82), while Azmi et al. [15] reported rare but severe cases in patients with diabetes using SGLT2is, warranting clinical caution.

Microbiologically, FG is consistently described as a polymicrobial infection involving synergistic aerobes and anaerobes [17], [18]. Escherichia coli was the most common isolate, but had a relatively lower mortality rate (25.9%) than Candida albicans (61.1%) and other pathogens such as Proteus, Pseudomonas, and Acinetobacter spp. [4]. Fungal infections, although rare, were associated with the highest mortality (up to 68.2%) and may reflect delayed treatment or immunosuppression [16], [18]. The polymicrobial synergy contributes significantly to tissue destruction. Therefore, early culture collection and prompt initiation of broad-spectrum empiric antibiotics, followed by targeted therapy, are critical components of early FG management [18], [20].

### 3.2 Treatment of FG

The management of FG is consistently described as a multimodal emergency, centered on three core interventions: early surgical debridement, empiric broad-spectrum antibiotics, and intensive supportive care [16], [18]. Early and aggressive surgery is universally regarded as the cornerstone of treatment and important for patients’ survival [18]. Shet et al. [16] showed that delays before surgery were linked to a higher mortality rate, as was the need for multiple surgeries, especially more than four debridement procedures. In nonsurgical management, mortality was extremely high in patients requiring mechanical ventilation (77.8%) or inotropic support (90.5%).

Empiric intravenous broad-spectrum antibiotics have to be initiated promptly and tailored based on cultures later on. Typical regimens include carbapenems or beta-lactam/beta-lactamase inhibitors with clindamycin or metronidazole [15], [16], [18]. Supportive care (fluid resuscitation, glycemic control, and hemodynamic stabilization) is essential, with some patients requiring intensive care unit management [16], [17].

Further adjunctive interventions such as diversional stomas (eg, colostomies) were linked to higher mortality and costs, likely reflecting a selection bias due to severe disease. Thus, their individualized use is recommended in cases with extensive perianal involvement or sphincter damage [16], [19].

Hyperbaric oxygen (HBO) therapy was associated with reduced mortality (16.6% vs 25.9%). While promising, HBO therapy is limited by cost and availability, and should be considered only as an adjunct in selected high-risk patients without delaying surgery [20].

Reconstructive surgery was detailed by Susini et al. [17], who reviewed 576 procedures. Minimally invasive methods (eg, grafts and local flaps) were used in 77.6% of cases, while locoregional flaps were reserved for more extensive defects. The authors advocate for early multidisciplinary planning to optimize outcomes.

Lastly, both Silverii et al. [21] and Azmi et al. [15] reviewed patients with FG on SGLT2is. While Silverii et al. [21] found no significant increase in risk in a meta-analysis, Azmi et al. [15] reported rare but severe cases in which SGLT2i therapy was discontinued at diagnosis and replaced by other antidiabetic drugs, and patients proceeded with standard management (aggressive debridement, antibiotics, fluid resuscitation, incisions, drainage, insulin, etc.).

### 3.3 Outcomes of FG

Despite advances in multimodal care, FG carries high mortality and morbidity. Reported mortality rates vary due to differences in patient profiles, microbial etiology, and treatment timing. Functional outcomes and quality of life depend on disease extent, number of surgeries, and reconstructive access. The most comprehensive mortality data shown by Shet et al. [16] reported a pooled mortality rate of 20.4% across 3646 patients. Mortality was highest among HIV-positive (54.2%) and fungal-infected (68.2%) patients, although sample sizes were limited. Female patients, while affected less frequently, had disproportionately higher mortality in several reviews [16], [17].

Early debridement is supposed to be connected to better outcomes, with Lewis et al. [18] pointing out that diagnostic delays should be avoided.

Patients being treated with a diversion stoma showed significantly higher mortality (OR 1.71, 95% CI 1.13–2.59, *p* = 0.01), more procedures, and longer hospital stays. Thus, clinical decision-making should be individualized, with particular attention to disease burden and anal sphincter integrity [19].

Long-term reconstruction outcomes, detailed by Susini et al. [17], showed that 77.6% of procedures used minimally invasive methods (eg, skin grafts and secondary intention healing), while 22.4% involved locoregional flaps. No free flaps were reported. Early multidisciplinary planning was associated with favorable aesthetic and functional results.

HBO therapy, reviewed by Schneidewind et al. [20], showed a lower pooled mortality rate (16.6%) than standard care (25.9%). Despite potential benefit, data heterogeneity and bias limit generalizability. HBO therapy may be considered in selected cases but should not delay surgery.

Regarding SGLT2is, Silverii et al. [21] found no increased FG risk in a meta-analysis of 84 randomized controlled trials (Mantel-Haenszel OR 0.41). Azmi et al. [15], reporting on 14 case reports, noted that all patients survived after drug discontinuation and standard treatment, although the small sample precludes firm conclusions.

### 3.4 Risk of bias assessment of the included systematic reviews

The rating of the overall confidence in the results of the included reviews is moderate (Table 2). This rating was mainly due to the following critical domains in the AMSTAR2 tool: justification for excluding individual studies and risk of bias from individual studies being included in the review.

**Table 2:** Overall confidence of the results of the included reviews using AMSTAR2 (*n* = 7)

Reference	Rating the overall confidence in the results of the review
Azmi et al. (2025) [15]	Moderate
Shet et al. (2024) [16]	Moderate
Susini et al. (2024) [17]	Moderate
Lewis et al. (2021) [18]	Moderate
Sarofim et al. (2021) [19]	Moderate
Schneidewind et al. (2020) [20]	Moderate
Silverii et al. (2020) [21]	Moderate

## 4 Discussion

To the best of our knowledge, we conducted the first umbrella review concerning the standard of care for FG to provide the best evidence for clinical practice and for planning further comprehensive research.

### 4.1 Diagnosis and risk factors of FG

The clinical diagnosis of FG remains challenging due to nonspecific early signs. The most consistently reported risk factors include diabetes mellitus, immunosuppression (particularly HIV), obesity, chronic organ dysfunction, and possibly female sex. While SGLT2i use does not appear to confer a statistically significant increase in risk, case-based evidence suggests heightened vigilance. Risk stratification based on these factors is essential to facilitate early recognition, appropriate imaging, and timely initiation of definitive surgical management.

### 4.2 Treatment of FG

Effective FG treatment hinges on early surgical debridement, prompt initiation of broad-spectrum antibiotics, and robust critical-care support [16], [17], [18]. The use of stomas and HBO therapy remains controversial and case dependent, while reconstructive surgery is best individualized based on wound characteristics and patient recovery [17], [19], [20]. Thereof, there is consensus that standardization of treatment algorithms and multicenter trials are needed to optimize care pathways in this high-mortality condition [16], [17].

### 4.3 Outcomes of FG

Mortality associated with FG remains high, although estimates vary across systematic reviews due to differences in populations, severity, and study designs. The largest synthesis by Shet et al. [16] reported a pooled mortality rate of 20.4% across 3646 patients, with subgroup analyses identifying a significantly higher risk. These findings highlight the importance of host factors and infectious burden in shaping the prognosis.

Patients receiving diversion stomas also reported a higher mortality rate, although the authors noted that this likely reflects more advanced disease rather than a harmful effect of the procedure itself [19]. By contrast, Schneidewind et al. [20] reported a reduction in mortality rate in patients treated with HBO therapy, suggesting a potential survival benefit from adjunctive treatment, although data were limited by study heterogeneity and quality.

Although reconstructive surgery appears safe and effective, especially after infection control, the evidence is limited by retrospective design, small sample sizes, and missing follow-up data. These call for prospective studies with standardized outcome reporting [17].

Reviews of FG in the context of SGLT2is were limited by under-reporting and lack of clinical details. While FG is a rare adverse event, these reviews highlight the importance of clinician awareness and timely management. A causal relationship remains unproven due to limited and heterogeneous evidence. These findings are limited by potential publication and reporting biases.

Overall, despite high acute-phase mortality, especially in high-risk subgroups, outcomes improve markedly with early diagnosis and comprehensive surgical and supportive care.

### 4.4 Limitations of this umbrella review

Naturally, our work is not without limitations as well. First, we were able to provide only moderate confidence mainly due to the risk of bias from the individual studies being included in the reviews. The included studies mainly have a retrospective study design with small sample sizes and therefore a high risk of selection bias. Second, the included reviews did not report some major aspects of FG, for example, FG severity index [22]. Third, we did not perform a hand search of target journals or conference proceedings. Lastly, some of the included reviews did not report proper statistics or all relevant statistical information, so 95% CIs were not reportable or calculable from the data available to us [15], [16], [17], [18], [20]. Despite these obvious limitations, we summarized the current evidence for the standard of care of FG very comprehensively and identified the knowledge gaps, which is essential to plan further robust research.

### 4.5 Outlook

Unfortunately, it is well known that research and improvement of treatment of rare diseases with low incidence rates are a problem in health care. In this setting, even planning of prospective clinical studies is challenging. One approach to solve that problem is the European Reference Network eUROGEN for rare urogenital diseases and complex conditions in both children and adults [4], [23]. For this reason, an online platform with registry study is the right approach to tackle FG and improve outcomes for our patients [4]. In our opinion, research should focus on shortening the time to diagnosis and treatment, further standardizing care in FG and establishing guidelines, evaluating adjunct therapies for special patient groups (eg, HBO therapy), and raising awareness of this rare disease [4]. Additionally, there might be discipline-specific responsibilities, for example, colostomies for general surgery, urinary diversion for urology, complex reconstruction for plastic surgery, or care of female patients for gynecology. These aspects should also be addressed in further research.

## 5 Conclusions

Across the seven reviews mentioned, FG remains a life-threatening condition with substantial mortality.

However, survival improves markedly with early diagnosis, repeated surgical debridement, and multidisciplinary care. Adjunctive therapies, such as HBO therapy, may reduce mortality further, although evidence is limited. In survivors, reconstructive outcomes are favorable, with no reported deaths after reconstruction. FG associated with the use of SGLT2is appears rare but serious; outcomes are generally positive when treated promptly. Overall, the literature emphasizes the need for early intervention, risk stratification tools, and standardized outcome reporting to guide future clinical decision-making and, as we mentioned above, further comprehensive research. In this context, a prospective registry study might be the most suitable and robust study design.

  ***Author contributions*:** Laila Schneidewind had full access to all the data in the study and takes responsibility for the integrity of the data and the accuracy of the data analysis.

  *Study concept and design*: Aschwanden, Fankhauser, Schneidewind.

*Acquisition of data*: Aschwanden, Wimmer, Schneidewind.

*Analysis and interpretation of data*: All authors.

*Drafting of the manuscript*: Müssgens, Schneidewind.

*Critical revision of the manuscript for important intellectual content*: All authors.

*Statistical analysis*: Müssgens, Schneidewind.

*Obtaining funding*: None.

*Administrative, technical, or material support*: All authors.

*Supervision*: Fankhauser, Schneidewind.

*Other*: None.

  ***Financial disclosures:*** Laila Schneidewind certifies that all conflicts of interest, including specific financial interests and relationships and affiliations relevant to the subject matter or materials discussed in the manuscript (eg, employment/affiliation, grants or funding, consultancies, honoraria, stock ownership or options, expert testimony, royalties, or patents filed, received, or pending), are the following: None.

  ***Funding/Support and role of the sponsor*:** None.
